# Supplementary material for: Digital Media Use in Association with Sensory Taste Preferences in European Children and Adolescents—Results from the I.Family Study
Source: Foods. 2021 Feb 9;10(2):377. doi: 10.3390/foods10020377 (PMC7916161; doi:10.3390/foods10020377)
Supplement: Supplementary file 1 [file foods-10-00377-s001.pdf]

Digital media use in association with sensory taste preferences in European children and adolescents- results from the I.Family study

Elida Sina[1]

[1] Leibniz Institute for Prevention Research and Epidemiology-BIPS, Bremen, Germany

Online Supplementary Material

Figure S1. Participant flow chart from I.Family study included in current analyses.

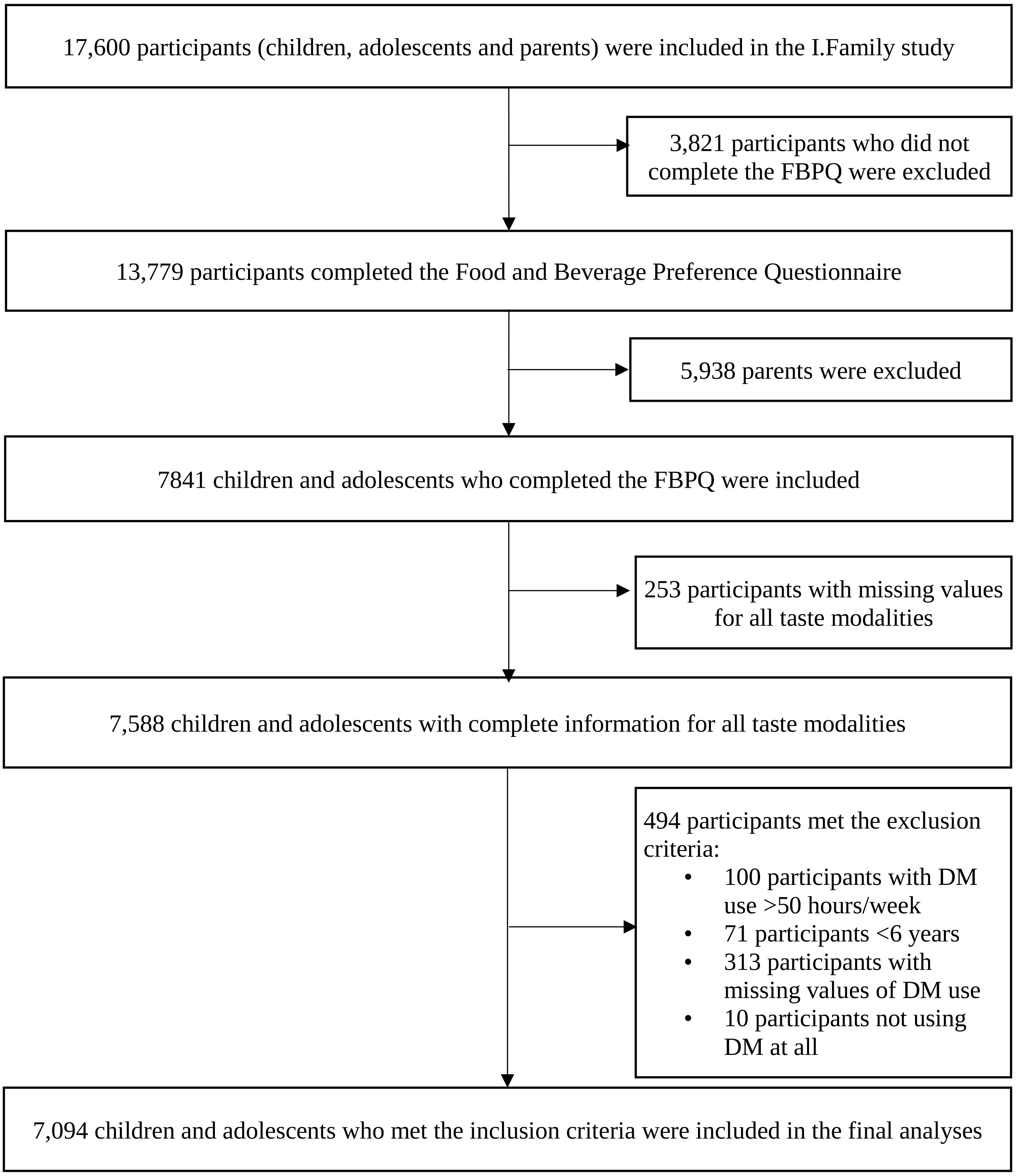

Table S1. Duration of media use in European children and adolescents, by sex and age group[1]

	Age group								All	
	Children				Adolescents					
	Males		Females		Males		Females			
	**n**	**%**	**n**	**%**	**n**	**%**	**n**	**%**	**N**	**%**
**All**	2026	28.6	1986	28.0	1476	20.8	1606	22.6	7094	100.0
**Total DM exposure**										
≤1 h/day	284	4.0	420	5.9	67	0.9	159	2.2	930	13.1
1 to 2 h/day	733	10.3	801	11.3	317	4.5	427	6.0	2278	32.1
2 to 3 h/day	541	7.6	468	6.6	374	5.3	426	6.0	1809	25.5
>3 h/day	468	6.6	297	4.2	718	10.1	594	8.4	2077	29.3
**TV viewing**										
Not at all	8	0.1	5	0.1	19	0.3	10	0.1	42	0.6
≤1 h/day	767	10.8	857	12.1	420	5.9	557	7.9	2601	36.7
1 to 2 h/day	791	11.2	740	10.4	568	8.0	587	8.3	2686	37.9
2 to 3 h/day	389	5.5	322	4.5	378	5.3	350	4.9	1439	20.3
> 3 h/day	71	1.0	62	0.9	91	1.3	102	1.4	326	4.6
**PC use**										
Not at all	248	3.5	519	7.3	81	1.1	415	5.9	1263	17.8
<=1 h/day	1342	18.9	1301	18.3	658	9.3	861	12.1	4162	58.7
1 to 2 h/day	302	4.3	126	1.8	419	5.9	191	2.7	1038	14.6
2 to 3 h/day	100	1.4	21	0.3	205	2.9	85	1.2	411	5.8
> 3 h/day	15	0.2	2	0.0	83	1.2	27	0.4	127	1.8
Missing	19	0.3	17	0.2	30	0.4	27	0.4	93	1.3
**Internet exposure**										
Not at all	399	5.6	411	5.8	30	0.4	28	0.4	868	12.2
≤1 h/day	1164	16.4	1194	16.8	550	7.8	590	8.3	3498	49.3
1 to 2 h/day	225	3.2	210	3.0	358	5.0	369	5.2	1162	16.4
2 to 3 h/day	108	1.5	57	0.8	273	3.8	260	3.7	698	9.8
> 3 h/day	39	0.5	25	0.4	247	3.5	338	4.8	649	9.1
Missing	91	1.3	89	1.3	18	0.3	21	0.3	219	3.1
**Smartphone use**										

	Age group								All	
	Children				Adolescents					
	Males		Females		Males		Females			
	**n**	**%**	**n**	**%**	**n**	**%**	**n**	**%**	**N**	**%**
Not at all	1222	17.2	1146	16.2	328	4.6	254	3.6	2950	41.6
≤1 h/day	339	4.8	400	5.6	392	5.5	374	5.3	1505	21.2
1 to 2 h/day	155	2.2	140	2.0	276	3.9	285	4.0	856	12.1
2 to 3 h/day	47	0.7	50	0.7	178	2.5	231	3.3	506	7.1
> 3 h/day	30	0.4	46	0.6	228	3.2	401	5.7	705	9.9
Missing	233	3.3	204	2.9	74	1.0	61	0.9	572	8.1

[1]DM-Digital media, TV-television, PC-computer/game console

Association of media use with sweet taste preference, by sweets intake propensity

Table S2 and S3 show the results of the sensitivity analyses where we stratified by propensities to investigate whether the association of exposure to total media with sweet and fatty TP differs by the respective propensities. The fully adjusted model showed a positive trend in the association between exposure to different durations of DM and sweet TP (**Table S2**) in the high sweet propensity strata (e.g. DM exposure ≥3 h/day: OR=1.27; 95%CI=0.98-1.65). After stratification by sex and age (**Table S4**), we observed that exposure for >2 h/day to DM was associated with sweet TP across all groups, besides male children. In the low sweet propensity strata, we did not observe an association between different durations of DM exposure and sweet TP (**Table S2).** However, after further stratification by age and sex (**Table S4**), the association attenuated and was positive both in adolescents males (OR=1.23, 95%CI=0.86-1.76) and adolescent females (OR=1.29; 95%CI=0.96-1.73).

Table S2. Association of total digital media exposure with sweet taste preference in European children and adolescents, by sweet intake propensity[1, 2]

	**High sweet propensity (N=3454)**		**Low sweet propensity (N=3631)**	
	**Raw model**	**Adjusted model**	**Raw model**	**Adjusted model**
**Total DM exposure (**Ref. ≤1 h/day)	**Odds ratios (OR) and 95% Confidence limits (95%CI)**			
1-2 h/day	1.11 (0.87-1.42)	1.10 (0.86-1.42)	0.93 (0.76-1.14)	0.95 (0.77-1.17)
2-3 h/day	1.13 (0.88-1.45)	1.16 (0.89-1.50)	0.88 (0.71-1.09)	0.91 (0.72-1.14)
≥ 3 h/day	1.22 (0.96-1.56)	1.27 (0.98-1.65	1.00 (0.81-1.24)	1.06 (0.83-1.33)

[1]Logistic regression models were adjusted for sex, age group, snack consumption, HDAS, parental educational status, migrant background and country -OR not reported. [2] DM-Digital media

Association of media use with fatty taste preference, by fat intake propensity

The stratified analyses for the fatty taste preference (**Table S3**), showed an association with prolonged DM exposure in the high fat propensity group (e.g. DM exposure for ≥3 hours/day: OR=1.28, 95%CI=0.99-1.65) After stratification by age and sex (**Table S4**), the association was positive both in adolescent males and females (respectively, OR=1.18, 95%CI=0.78-1.81; OR=1.21; 95%CI= 0.85-1.72). In the low propensity strata (**Table S3**), a positive association was observed between different durations of DM exposure and fatty TP (e.g. DM exposure for ≥3 hours/day: OR=1.36; 95%CI=1.06-1.73). After stratification, being exposed for >2 hours/day to DM was associated with fatty TP in adolescents (males: OR=1.19; 95%CI=0.85-1.66; females: OR=1.45; 95%CI=1.09-1.92), compared to DM exposure of ≤2 hour/day.

Table S3. Association of total media exposure with fatty taste preference in European children and adolescents, by fat intake propensity[1,2]

	**High fat propensity (N=3483)**		**Low fat propensity (N=3607)**	
	**Raw model**	**Adjusted model**	**Raw model**	**Adjusted model**
**Total DM exposure (**Ref. ≤1 h/day)	**Odds ratios (OR) and 95% Confidence limits (95%CI)**			
1-2 h/day	1.20 (0.95-1.51)	1.20 (0.95-1.52)	1.05 (0.85-1.30)	1.14 (0.91-1.43)
2-3 h/day	1.06 (0.84-1.35)	1.07 (0.83-1.37)	0.99 (0.79-1.24)	1.18 (0.93-1.49)
≥ 3 h/day	1.26 (1.00-1.60)	1.28 (0.99-1.65	1.00 (0.80-1.24)	**1.36 (1.06-1.73)**

[1]Logistic regression models were adjusted for sex, age group, snack consumption, HDAS, parental educational status, migrant background and country -OR not reported. [2] DM-Digital media

Table S4. Association of total media exposure with sweet and fatty taste preference in European children and adolescents, by sex and age group [1,2]

	Adjusted Model			
	**Males**		**Females**	
	**<12 Years**	**≥12 Years**	**<12 Years**	**≥12 Years**
	**Odds ratios (OR) and 95% Confidence limits (95%CI)**			
**Sweet taste preference**	**High sweet propensity**			
	**N=961**	**N=803**	**N=907**	**N=783**
**Total DM exposure** (Ref. ≤2 h/day) > 2h/day	0.90 (0.67-1.21)	1.34 (0.95-1.90)	1.25 (0.92-1.70)	1.13 (0.81-1.58)
	**Low sweet propensity**			
	**N=1062**	**N=672**	**N=1075**	**N=822**
	0.91 (0.69-1.20)	1.23 (0.86-1.76)	0.92 (0.70-1.21)	1.29 (0.96-1.73)
**Fatty taste preference**	**High fat propensity**			
	**N=1121**	**N=671**	**N=1054**	**N=637**
**Total DM exposure** (Ref. ≤2 h/day) > 2h/day	0.87 (0.66-1.16)	1.18 (0.78-1.81)	1.05 (0.79-1.39)	1.21 (0.85-1.71)
	**Low fat propensity**			
	**N=903**	**N=805**	**N=931**	**N=968**
	0.81 (0.60-1.10)	1.19 (0.85-1.66)	1.14 (0.84-1.53)	**1.45** **(1.09-1.92)**

[1]Logistic regression models were adjusted for age (continuous), snack consumption, HDAS, parental educational status, migrant background and country -OR not reported. [2] DM-Digital media
